# Supplementary material for: The causal role of circulating inflammatory markers in osteoporosis: a bidirectional Mendelian randomized study
Source: Front Immunol. 2024 Jul 18;15:1412298. doi: 10.3389/fimmu.2024.1412298 (PMC11291241; doi:10.3389/fimmu.2024.1412298)
Supplement: Supplementary file 1 [file DataSheet_1.zip › supplementary material/OSTEOPOROSIS-CIM/GCST90274816.tsv.gz_leave_one_out.pdf]

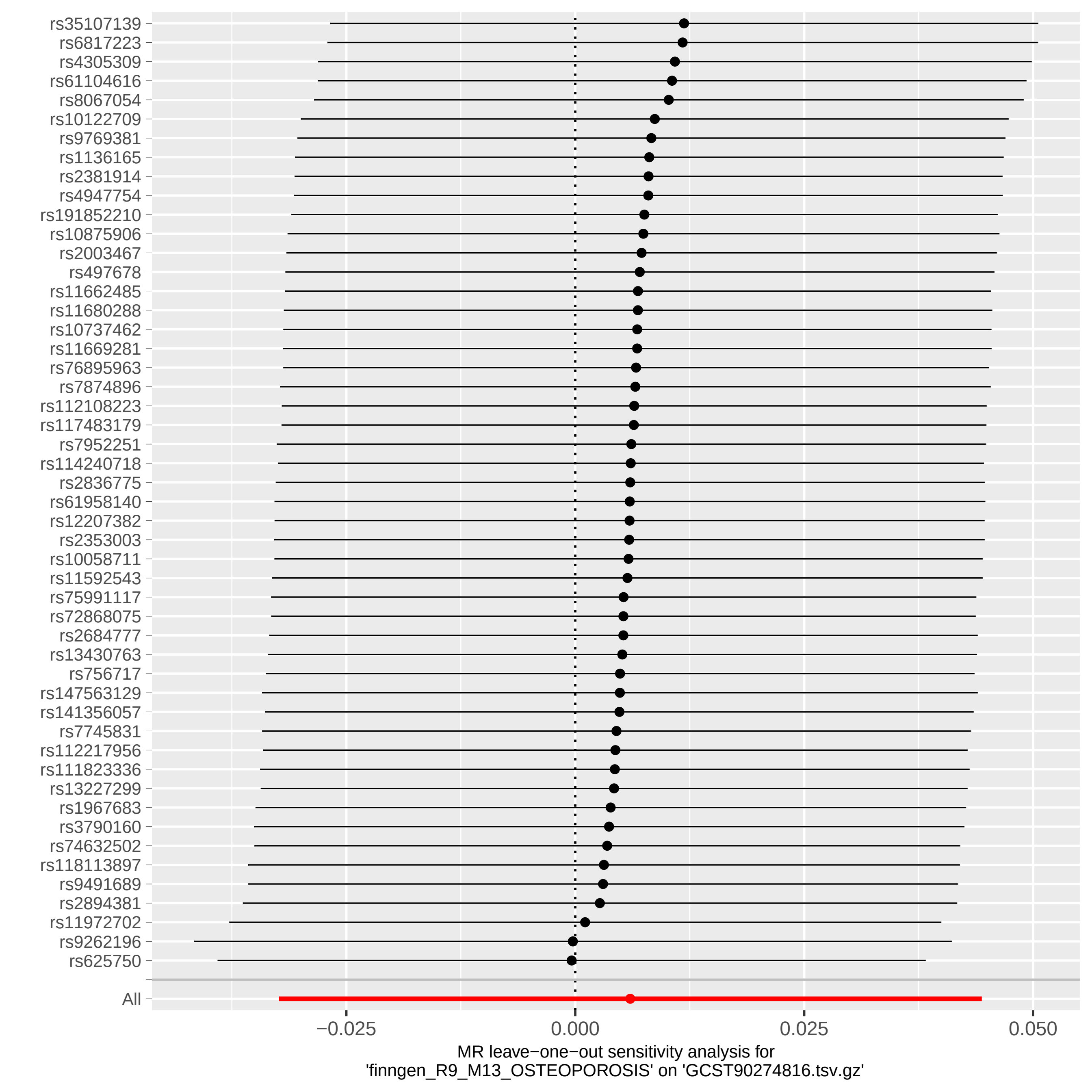
rs35107139
rs6817223
rs4305309
rs61104616
rs8067054
rs10122709
rs9769381
rs1136165
rs2381914
rs4947754
rs191852210
rs10875906
rs2003467
rs497678
rs11662485
rs11680288
rs10737462
rs11669281
rs76895963
rs7874896
rs112108223
rs117483179
rs7952251
rs114240718
rs2836775
rs61958140
rs12207382
rs2353003
rs10058711
rs11592543
rs75991117
rs72868075
rs2684777
rs13430763
rs756717
rs147563129
rs141356057
rs7745831
rs112217956
rs111823336
rs13227299
rs1967683
rs3790160
rs74632502
rs118113897
rs9491689
rs2894381
rs11972702
rs9262196
rs625750
All
−0.025
0.000
0.025
0.050
MR leave−one−out sensitivity analysis for
'finngen_R9_M13_OSTEOPOROSIS' on 'GCST90274816.tsv.gz'
